# Trophic Dynamics of Filter Feeding Bivalves in the Yangtze Estuarine Intertidal Marsh: Stable Isotope and Fatty Acid Analyses

**DOI:** 10.1371/journal.pone.0135604

**Published:** 2015-08-11

**Authors:** Sikai Wang, Binsong Jin, Haiming Qin, Qiang Sheng, Jihua Wu

**Affiliations:** 1 Coastal Ecosystems Research Station of the Yangtze River Estuary, Ministry of Education Key Laboratory for Biodiversity Science and Ecological Engineering, School of Life Sciences, Fudan University, Shanghai 200438, China; 2 Center for Watershed Ecology, Institute of Life Science, Nanchang University, Nanchang 330031, China; Instituto Español de Oceanografía, SPAIN

## Abstract

Benthic bivalves are important links between primary production and consumers, and are essential intermediates in the flow of energy through estuarine systems. However, information on the diet of filter feeding bivalves in estuarine ecosystems is uncertain, as estuarine waters contain particulate matter from a range of sources and as bivalves are opportunistic feeders. We surveyed bivalves at different distances from the creek mouth at the Yangtze estuarine marsh in winter and summer, and analyzed trophic dynamics using stable isotope (SI) and fatty acid (FA) techniques. Different bivalve species had different spatial distributions in the estuary. *Glauconome chinensis* mainly occurred in marshes near the creek mouth, while *Sinonovacula constricta* preferred the creek. Differences were found in the diets of different species. *S*. *constricta* consumed more diatoms and bacteria than *G*. *chinensis*, while *G*. *chinensis* assimilated more macrophyte material. FA markers showed that plants contributed the most (38.86 ± 4.25%) to particular organic matter (POM) in summer, while diatoms contributed the most (12.68 ± 1.17%) during winter. Diatoms made the largest contribution to the diet of *S*. *constricta* in both summer (24.73 ± 0.44%) and winter (25.51 ± 0.59%), and plants contributed no more than 4%. This inconsistency indicates seasonal changes in food availability and the active feeding habits of the bivalve. Similar FA profiles for *S*. *constricta* indicated that the bivalve had a similar diet composition at different sites, while different δ^13^C results suggested the diet was derived from different carbon sources (C_4_ plant *Spartina alterniflora* and C_3_ plant *Phragmites australis* and *Scirpus mariqueter*) at different sites. Species-specific and temporal and/or spatial variability in bivalve feeding may affect their ecological functions in intertidal marshes, which should be considered in the study of food webs and material flows in estuarine ecosystems.

## Introduction

Marshes with emergent vegetation are a common feature of most estuaries and coastal plains all over the world. Estuarine and coastal marshes have been widely recognized as crucial nursery grounds for many fish, birds and invertebrates [1, 2]. However, the complexity of physical and biological processes at land-sea transitional zones poses challenges for understanding trophic interactions and food web dynamics in this type of ecosystem. In many instances, the structure of the estuarine food web is complicated by seasonal changes resulting from freshwater discharge shifts, and by the spatial mosaic within estuaries created by different vegetation types and tidal amplitudes [3–6].

Benthic bivalves are important links between primary production and high level trophic consumers such as fishes, crabs and birds, and are key intermediates in the energy flow of estuarine systems. Bivalves are mainly benthic filter feeders. They obtain food by filtering out particulate material, and exert grazing control over phytoplankton production [7, 8]. However, the diet composition of bivalves in estuarine ecosystems is uncertain. As particulate materials in estuarine waters contain matter from a range of origins which are subject to a fluctuating and unpredictable environment, it is difficult to discriminate among food types using conventional techniques such as gut content analyses [9–12]. The issue is further complicated as bivalves are opportunistic feeders [13]. For instance, the clam *Macoma balthica*, has been reported to switch feeding guild (e.g., from suspension feeding to deposit feeding) depending on the availability of different foods [14, 15]. Doi et al. (2005) found that the diet of the bivalve *Corbicula japonica* varied along a stream gradient in northeastern Japan [16]. Bivalves inhabiting the upper stream took up food with a relatively high contribution of terrigenous material compared with bivalves in the Kitakami River mouth. Bachok et al. (2009) found that the diet of the bivalve, *Quidnipagus palatum* varied seasonally, at the Tomigusuku intertidal flat of Okinawa Island (Japan) [17]. The primary food of *Q*. *palatum* were vascular plants and bacteria from July to November, macroalgae and phytoplankton from November to January, and diatoms from January to July. Despite an increasing interest in food-web dynamics, little is known about the spatial and temporal changes of trophic pathways supporting bivalves in estuarine salt marshes.

Stable isotopes (SI) and fatty acid (FA) profiles have been widely used to investigate food sources of animal consumers, especially when the diet composition is difficult to evaluate using ordinary techniques such as gut content determination [18, 19]. For example, Rooker et al. (2006) used SI and FAs to determine the trophic structure and characterize carbon sources of juvenile and adult fishes associated with floating *Sargassum* in mid-shelf waters of the Gulf of Mexico [20]. Wang et al. (2015) analyzed the FAs and SI of zooplankton to assess their diets and estimate the proportional contribution of pelagic and sympagic carbon to their food sources [21]. SI and FA analyses can provide time integrated information on the assimilated diet of organisms [22, 23]. The relative trophic level of consumers can be identified using δ^15^N because the δ^15^N of a consumer is typically enriched by 3–4 ‰ relative to its diet, while δ^13^C provides information on assimilated carbon sources from primary producers [24–26]. FAs are carbon-rich compounds that are ubiquitous in all organisms, and can be used as biomarkers to trace the food sources of consumers in aquatic systems [27–29]. The fact that essential FAs are transferred from primary producers to higher trophic levels without change makes them suitable for use as biomarkers [22, 30]. SI and FA signatures thus serve as ideal complementary techniques to investigate food web dynamics.

This study aims to identify the spatial and temporal variation in the food composition of benthic filter feeding bivalves at estuarine intertidal marshes. The Yangtze River is ranked third, fourth, and fifth among the world’s rivers with regard to length, annual sediment flux, and water discharge to the sea, respectively. There are about 213 km^2^ salt marshes with well-developed creek systems in its estuary [31]. Characterized by an Asian monsoon climate, the estuary possesses distinct seasonal fluctuations in environmental conditions due to varying precipitation and hydrology. We surveyed the distribution of bivalve species at different distances along the Yangtze River estuarine marshes during winter and summer, and then analyzed their trophic dynamics using SI and FA techniques. This study addressed the following questions: (1) do different bivalve species differ in their distribution and dietary niches? (2) do bivalve diets shift between the summer wet period and the winter dry period? and (3) does the diet of bivalves change spatially along a gradient in the intertidal marshes?

## Materials and Methods

### Ethics statement

The study was carried out in a coastal wetland on Jiuduansha Island, Shanghai, permitted and managed by the Jiuduansha National Natural Reserve Administration. No specific permission was required to access this land, and field studies did not involve any endangered or protected species.

### Study site

**Fig 1:**
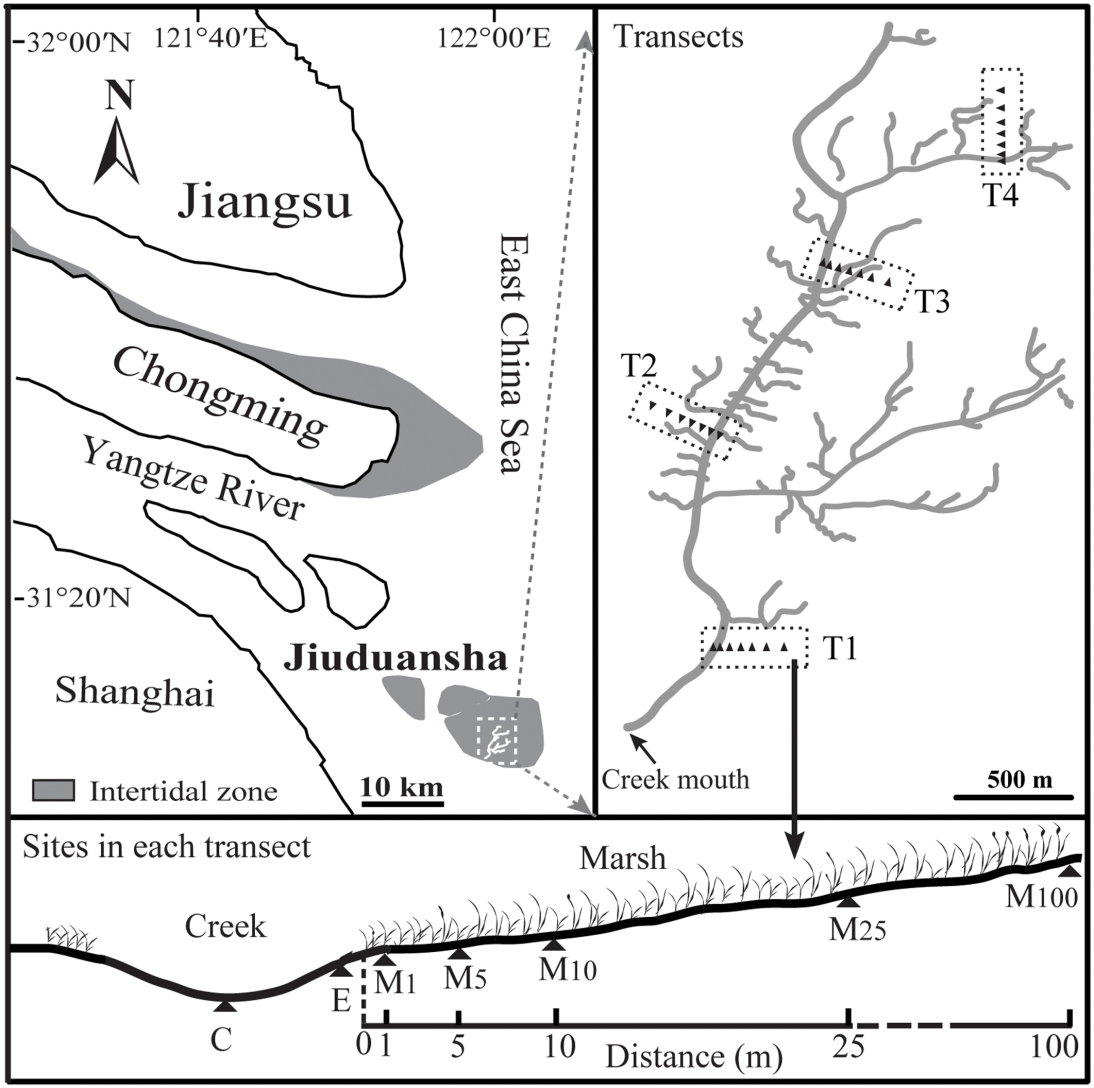
Location of the sampling sites in the Yangtze River estuary, China. T1, T2, T3 and T4 represent 4 transects with different elevation. The abbreviations of “C” and “E” represent the creek center and creek edge. Creek edge was defined as flat approximately 3 m extending into open water from the creek bank (the marsh-creek interface) [33].

The study sites were conducted in the Shanghai Jiuduansha Wetland National Nature Reserve (31°03'–31°38' N, 121°50'–122°05' E) of the Yangtze River estuary, China (Fig 1). The Jiuduansha wetlands are influenced by a sub-tropical monsoon climate. Mean annual precipitation is 1,145 mm, mainly falling during summer. Mean annual temperature is 15.7°C, with a mean temperature of the coldest and warmest months being 4.2°C in January and 27.3°C in July, respectively. The intertidal zones of the wetland are flooded by semi-diurnal meso-tides with a pronounced neap-spring inequality. Mean tidal range is 2.67 m, and the maximum tidal range is 4.62 m. Salinity is higher in winter than in summer due to variable precipitation and freshwater runoff of the Yangtze River from the dry season (November–January) to the wet season (July–September) [32]. Two native C_3_ plants, *Scirpus mariqueter* and *Phragmites australis* dominated the marshes before the exotic C_4_ plant *Spartina alterniflora* was introduced.

### Sampling design

#### Bivalve species distribution

Bivalves were surveyed during the wet summer season (July) and the dry winter season (November), 2008. Sampling was conducted along four transects (T1–T4) which were orientated perpendicular to direction of the tidal creek, each running from the center of the creek and extending into the adjacent marsh (Fig 1). Along each transect, there were two sample sites in the creek (one in the center and one at the edge) and five sampling sites in the marsh at distances of 1, 5, 10, 25 and 100 m from the creek bank. Elevation above sea level and dominant plant communities at each sampling site are listed in S1 Table. At each site five replicate sediment cores of 176.6 cm^2^ (diameter = 15 cm) were collected at 5 m intervals, up to a depth of 20 cm. The sediment cores were washed through a 0.5 mm mesh sieve. In the laboratory, all bivalve species were picked out, counted and identified.

#### Sampling for stable isotope (SI) and fatty acid (FA) analyses

We surveyed bivalves in 2008 and found that different bivalve species distribute at different sites. Then we raised a question if the bivalves at different sites have different dietary composition. Therefore, we conducted the trophic experiments in 2010 and 2011. Bivalves, sediment organic matter (SOM), suspended particulate organic matter (POM), and plant materials were collected during the summer wet period (July, 2010) and the winter dry period (January, 2011) for isotope and FA analyses. *Sinonovacula constricta* were collected from the creek center at each of the four transects, and *Glauconome chinensis* were mainly collected from the marsh sites at transect T1. FAs were not analyzed in samples of *G*. *chinensis* collected during the winter, due to inadequate sample quantities.

SOM was collected from each site by scraping approximately 1–2 mm from the surface of sediments during low tide. Three replicate SOM samples were collected from each site at 5 m intervals. POM was sampled from the center of the creek at each of the four transects by collecting 2 L water and then filtering the water through pre-combusted (450°C for 12 hours) Whatman GF/F glass fiber filters (47 mm filters) using a vacuum system.

Leaves of *Phragmites australis*, *Scirpus mariqueter* and *Spartina alterniflora* were collected simultaneously. Before SI measurement, all plant samples were cleaned with distilled water and scraped gently by hand to remove attached detritus.

All collected samples were put in an ice box after collection in the field and transported to the laboratory by car, within 1 hour. Bivalves were kept in filtered seawater overnight for gut-content clearance and then opened for tissue samples. All samples including bivalve tissue, SOM, POM and plant material were placed into the freeze dryer for overnight lyophilization, and then ground into a fine, homogeneous powder using a mortar and pestle for isotope and FA analyses.

#### Stable isotope (SI) analyses

All samples used for SI analyses were divided into two parts. One part was soaked in 1.2 N HCl for 4 to 5 min to eliminate carbonates [34], and then dried and ground for isotopic carbon analyses. The other part was for isotopic nitrogen analyses. This part received no acid treatment as this has been reported to affect the δ^15^N values [35]. Samples were weighed in a tin capsule and analyzed using an isotope ratio mass spectrometer (Delta plus Advantage, Thermo Scientific) at the Stable Isotope Laboratory for Inorganic Analysis and Testing Center, Institute of Geography and Limnology, Chinese Academy of Sciences, China. Isotope values are expressed in the δ unit notation as deviations from standards (Vienna Pee Dee Belemnite for δ^13^C and atmospheric N_2_ for δ^15^N) using the formula δ^13^C or δ^15^N = [(*R*_sample_/*R*_standard_)-1] × 10^3^, where *R* is ^13^C/^12^C or ^15^N/^14^N. The analytical precision of the measurements was < 0.1 ‰ and < 0.2 ‰ for carbon and nitrogen, respectively.

#### Fatty acid (FA) analyses

Total lipids were extracted from samples with chloroform:methanol (2:1, v/v). Esters of the FAs were prepared by transesterification with 14% BF_3_–MeOH for 1 h at 80°C. The FAMEs (FA methyl esters) obtained were analyzed using a Hewlett-Packard 7890A GC (Agilent Technologies, Santa Clara, CA, USA) equipped with an automatic sampler. All FAME samples were run in splitless mode, with a 1 μl injection per run at an injector temperature of 250°C using a DB-225 capillary column (30 m × 0.25 mm internal diameter, 0.25 μm film thickness, Agilent J & W; Agilent Co., USA) at a helium flow rate of 1.0 ml min^−1^. For further detail refer to Wang et al. (2014).

Fatty acid methyl esters (FAMEs) were identified by comparing the retention times of samples with the retention times of known standards (37 component FAME mix, Supelco 47885-U, Sigma Aldrich Inc., St. Louis, MO, USA), and were quantified by comparing peak areas with the area of the total identified FAs. The analysis was conducted using Agilent MSD Productivity ChemStation software. The abbreviated FA nomenclature used in this study was A:BnX, where A is the number of carbon atoms, B is the total number of double bonds, and X is the position of the double bond closest to the terminal methyl group.

We used some essential FAs as biomarkers which were identified from the published literature and considered as a specific group (vascular plants, bacteria, diatoms, dinoflagellates and zooplankton) or ubiquitous markers [36, 37]. FAs of vascular plants include 18:2n6, 18:3n3 [27, 38]. Bacterial FAs are those with odd-numbered carbon chains and branches, including 15:0, 17:0 and 18:1n7 [38, 39]. The FAs 14:0, 16:1 and 20:5n3 are common diatom markers, while 16:4n3 and 22:6n3 are used together as dinoflagellate markers [28, 40]. The FAs 20:1 and 22:1 are zooplankton markers [30, 41]. The FAs 16:0, 18:0 and 18:1n9 are considered as ubiquitous markers [36, 40]. FA results were expressed as the percentage of a specific FA marker relative to the sum of all FAs.

### Statistical analyses

Repeated measure analysis of variance (ANOVA) was used to test for spatial and temporal variations in the density of bivalves across transects and seasons. Two-way ANOVA with post-hoc Tukey tests were used to examine differences in δ^13^C and δ^15^N isotopic values between bivalve species (*Sinonovacula constricta* and *Glauconome chinensis*) and sampling seasons (summer and winter). One-way ANOVA with post-hoc Tukey tests were used to test for differences in the proportion of FA markers between bivalve species (*S*. *constricta* and *G*. *chinensis*). Differences in the proportions of FA markers in *S*. *constricta* between summer and winter were tested using Student *t*-tests. MANOVA with sigma-restricted parameterization was used to test for differences in FA marker percentages, among transects and seasons. Differences were regarded as significant at *P* < 0.05. All data were checked for normality (Shapiro-Wilk test) and homogeneity of variances (Bartlett and Levene test) prior to parametric analyses. Where necessary, data were square-root or log(x+1) transformed prior to analysis. Statistical analyses were conducted using STATISTICA software (StatSoft Inc., 2007, version 8.0, www.statsoft.com). Multivariate differences in FA profiles for all samples were analyzed using non-metric multidimensional scaling (n-MDS), based on the Bray-Curtis similarity index on log-transformed [log(x+1)] unstandardized data. Analyses were performed using Primer 5.0 (Primer-E Ltd., Plymouth, UK).

## Results

### Distribution pattern and food sources of different bivalve species

Two bivalve species *Glauconome chinensis* and *Sinonovacula constricta* were collected. *Glauconome chinensis* showed higher mean density in winter dry period than in summer, on the contrary, *S*. *constricta* showed higher density in summer than in winter. But, there were no significant effects of season on the density of each bivalve species statistically (ANOVA, repeated measures, *P* > 0.05). In both wet and dry seasons, the total density of bivalves increased in transects closer to the creek mouth.

**Fig 2:**
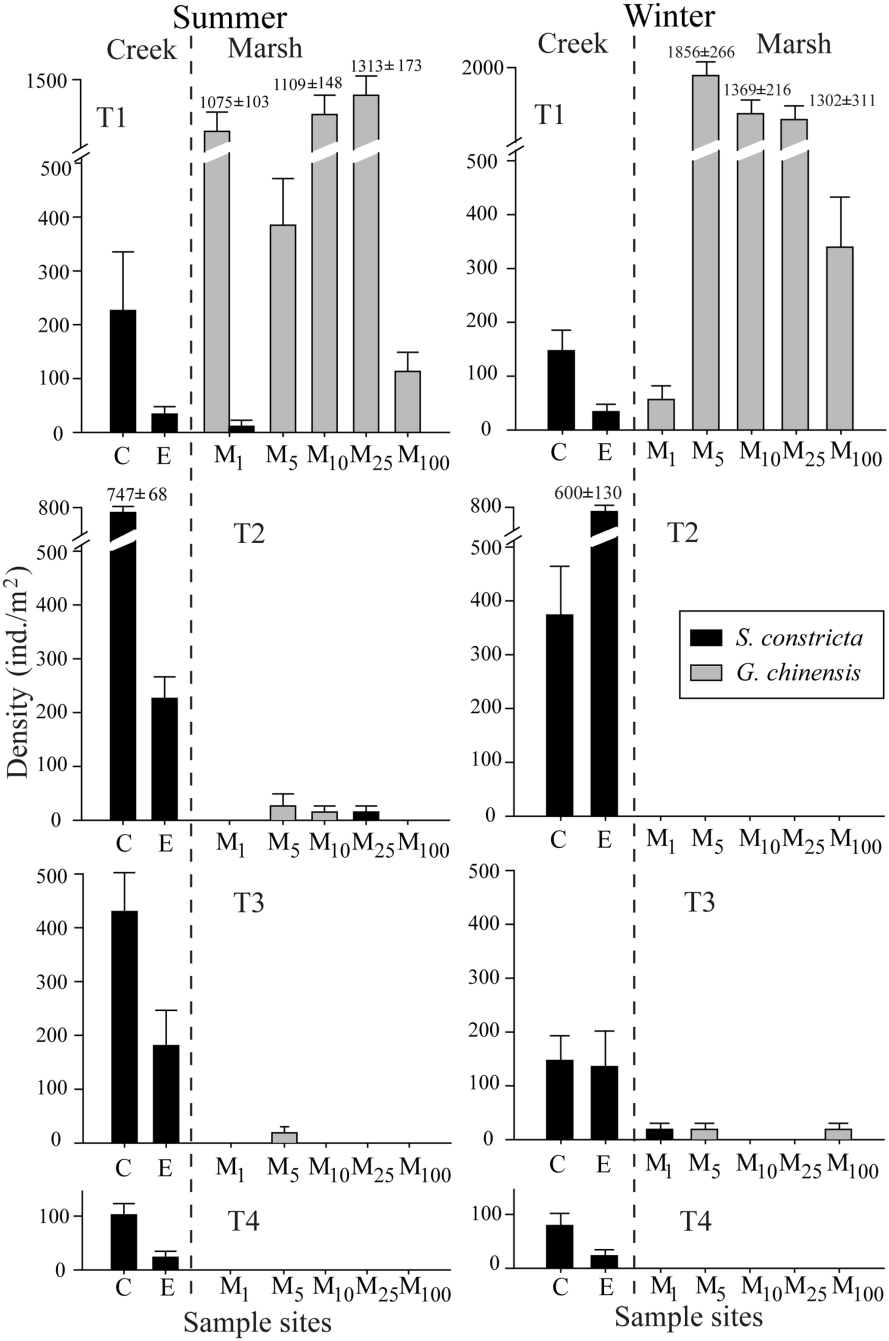
Density of bivalves (*Glauconome chinensis*, *Sinonovacula constricta*) at four transects (T1, T2, T3, T4) in summer and winter.

Two bivalve species showed different spatial distribution patterns, and significant site effect were found for the density of bivalves. *Glauconome chinensis* mainly occurred in marsh habitats, while *S*. *constricta* preferred creek habitats (Fig 2). *Glauconome chinensis* was the most abundant bivalve species, reaching a density of 1856 ± 266 ind. m^-2^ at site M5 of transect T1 in winter (Fig 2). *Sinonovacula constricta* had the highest abundance at transects T2 and T3.

**Fig 3:**
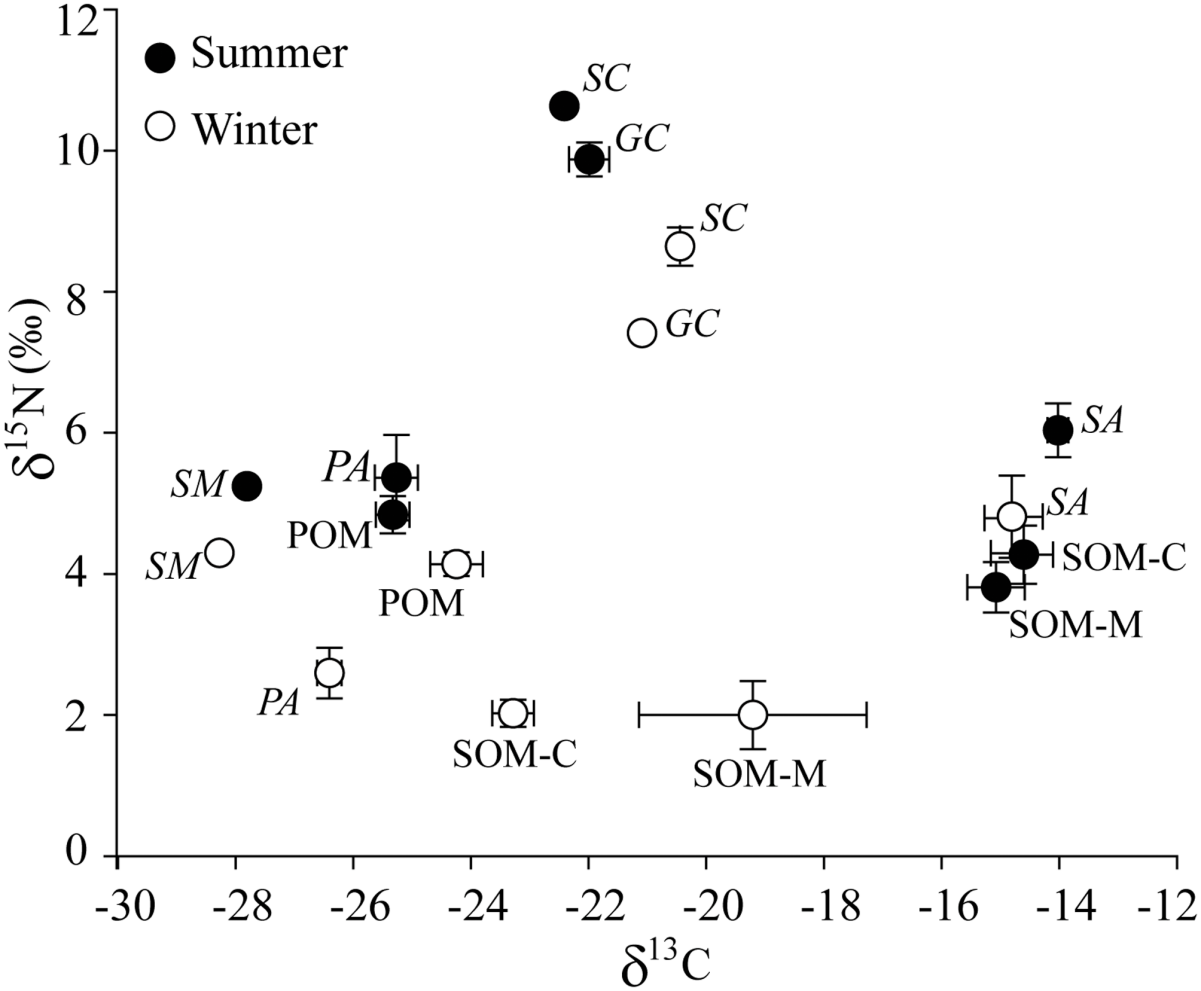
The dual isotope plot of δ^13^C and δ^15^N signatures (mean ± SE) of food sources and bivalves. *SC and GC* represent two bivalve species *Sinonovacula constricta* and *Glauconome chinensis*, respectively. *SM*, *PA and SA* represent three plant species of *Scirpus mariqueter*, *Phragmites australis* and *Spartina alterniflora* respectively. POM: particular organic matter. SOM: sediment organic matter. SOM-C and SOM-M represent SOM from the creek and the marshes respectively. Samples taken in summer or winter are indicated with filled or open symbols.

The δ^15^N values for *S*. *constricta* (10.63 ± 0.06 ‰ in summer; 8.64 ± 0.13 ‰ in winter) were significantly higher than the values for *G*. *chinensis* (9.87 ± 0.14 ‰ in summer; 7.41 ± 0.09 ‰ in winter) in both summer and winter (Tukey HSD test, *P* < 0.05) (Fig 3). In summer, there were no significant differences in δ^13^C values between *S*. *constricta* (−22.09 ± 0.14 ‰) and *G*. *chinensis* (−21.99 ± 0.23 ‰). In winter, however, *S*. *constricta* had a significantly enriched δ^13^C value (−20.42 ± 0.12 ‰) compared with *G*. *chinensis* (−21.09 ± 0.07 ‰) (Tukey HSD test, *P* < 0.05).

**Fig 4:**
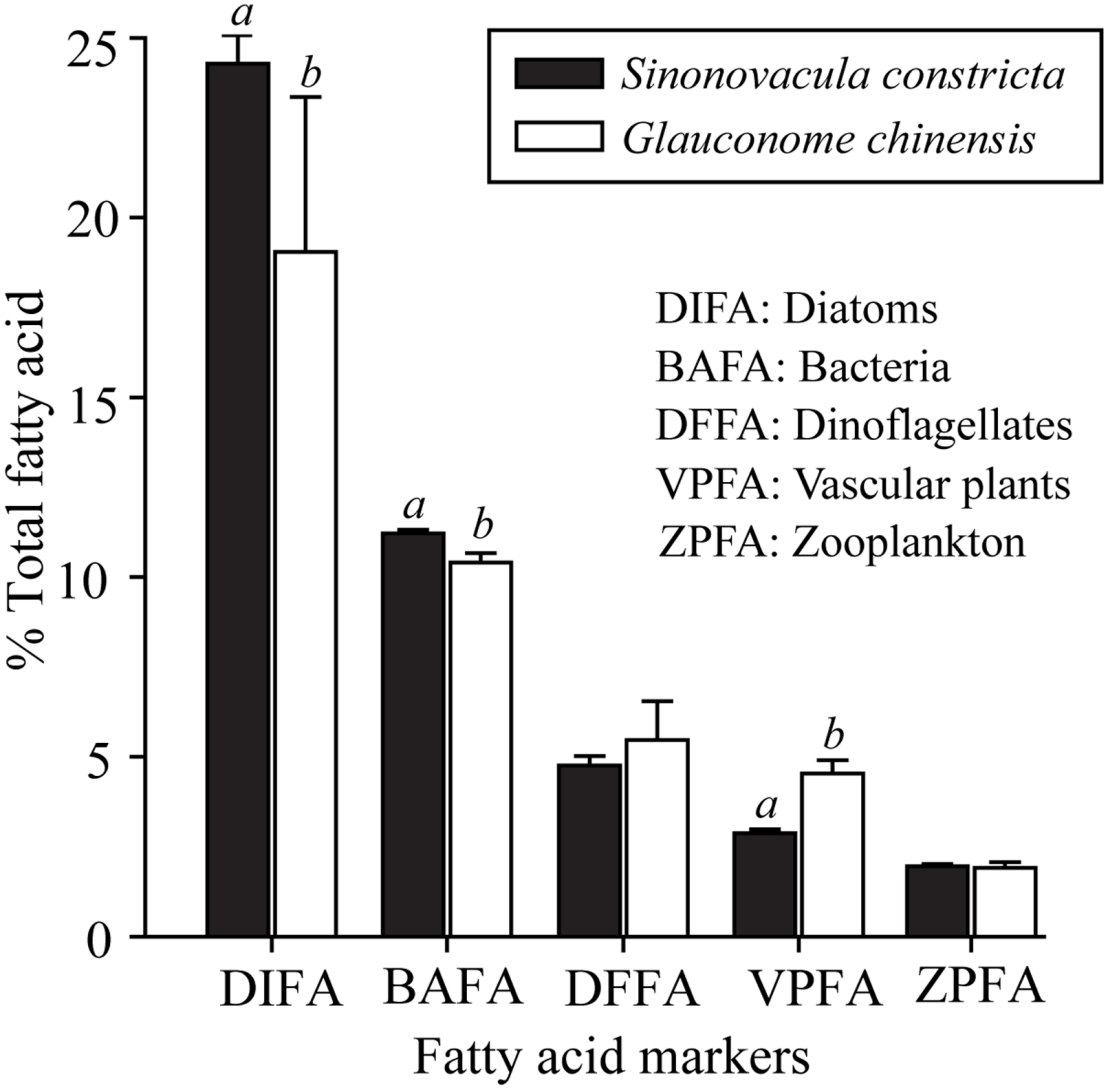
Relative percentages of fatty acid markers (diatom, bacteria, dinoflagellates, vascular plants and zooplankton) from *Sinonovacula constricta* and *Glauconome chinensis* in summer. Different superscript letters (*a*, *b*) refer to significant differences between two bivalve species (Tukey HSD test, *P* < 0.05).

During the wet summer season, the relative contributions of diatoms and bacterial FA markers, were found to be significantly higher in *S*. *constricta* (24.73 ± 0.44% and 10.95 ± 0.12%, respectively) compared with *G*. *chinensis* (19.52 ± 2.74% and 9.93 ± 0.47%, respectively) (Tukey HSD test, *P* < 0.05) (Fig 4). The percent contribution to FA markers from vascular plants was significantly higher in *G*. *chinensis* (4.88 ± 0.43%) compared with *S*. *constricta* (3.23 ± 0.09%). Both SI and FA marker analyses indicated that different bivalve species have different food sources.

### Temporal differences in stable isotopes and fatty acids

#### Stable isotopes (SIs)

The δ^13^C values of bivalves were in the range of potential sources include vascular plants, SOM and POM. Plants and SOM showed were more enriched δ^13^C values in summer than in winter (Fig 3). However, both bivalve species and POM showed an opposite pattern, more depleted δ^13^C values in summer than in winter. The consistent temporal variation in δ^13^C values of bivalves and POM may indicate that POM was the main food item of bivalves. In addition, the δ^15^N values of *G*. *chinensis* were 3.27 and 5.04 ‰, while *S*. *constricta* were 4.50 and 5.79 ‰ more enriched than POM in winter and summer respectively. The δ^15^N values for all bivalves and their potential food sources, and the δ^13^C values for plants and SOM, were more enriched in summer than in winter.

#### Fatty acids (FAs)

**Fig 5:**
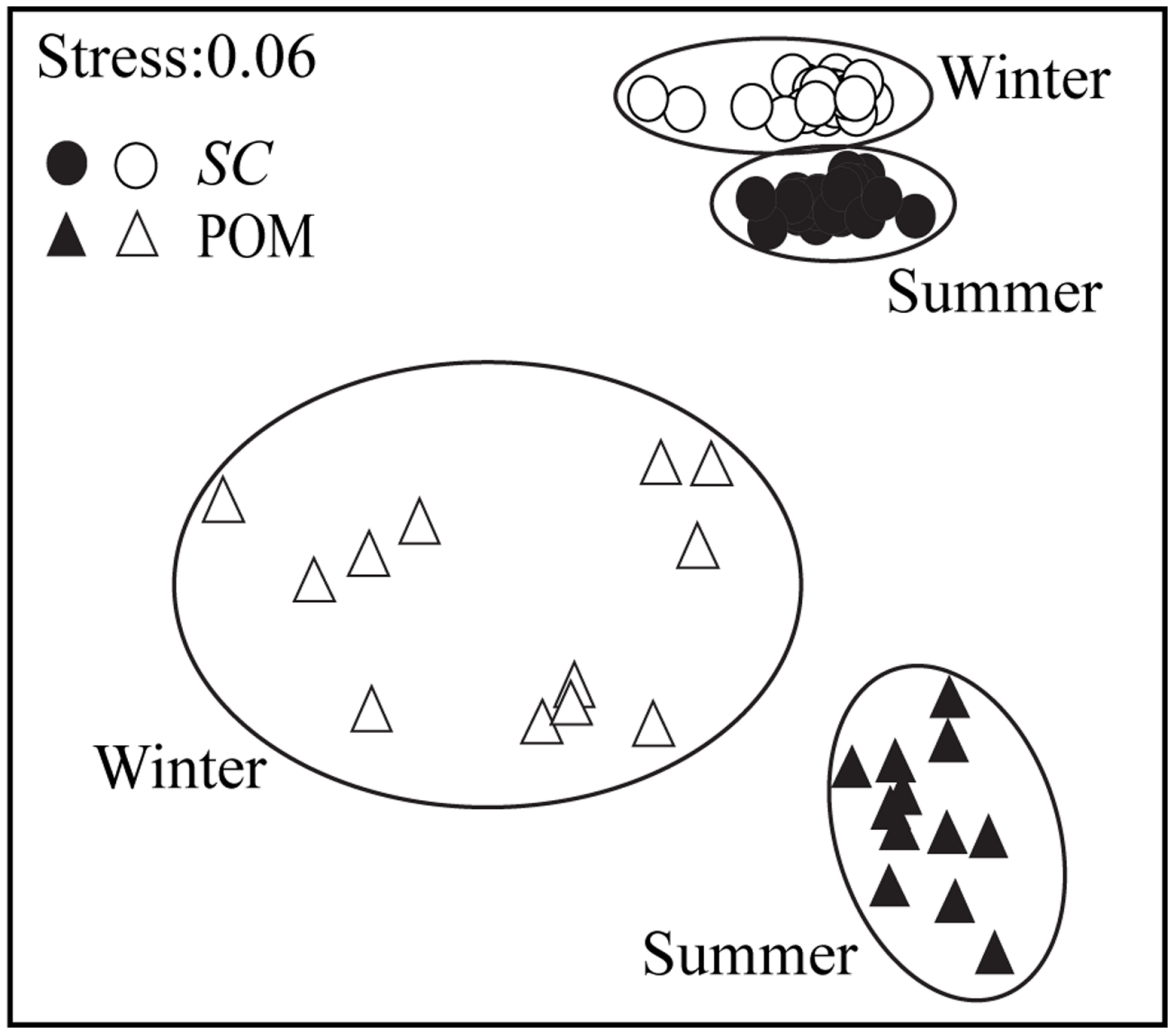
The MDS (Non-metric Multi-dimensional Scaling) ordination of fatty acid profiles of bivalve and particular organic matter in summer and winter. SC: *Sinonovacula constricta*. POM: particular organic matter. Samples in summer or winter are indicated with filled or open symbols.

Of the 30 FAs identified, 14:0, 16:0, 16:1, 16:4n3, 18:1n9, 18:2n6 and 18:3n3 accounted for 56–93% of total FAs (S2 Table). All analyzed materials including food sources and bivalve consumers were rich in 16:0 (17.55–37.61%). The MDS plot based on FA profiles (plotted as a % of total FAs) showed clear separations between seasons for both *S*. *constricta* and POM (Fig 5).

**Fig 6:**
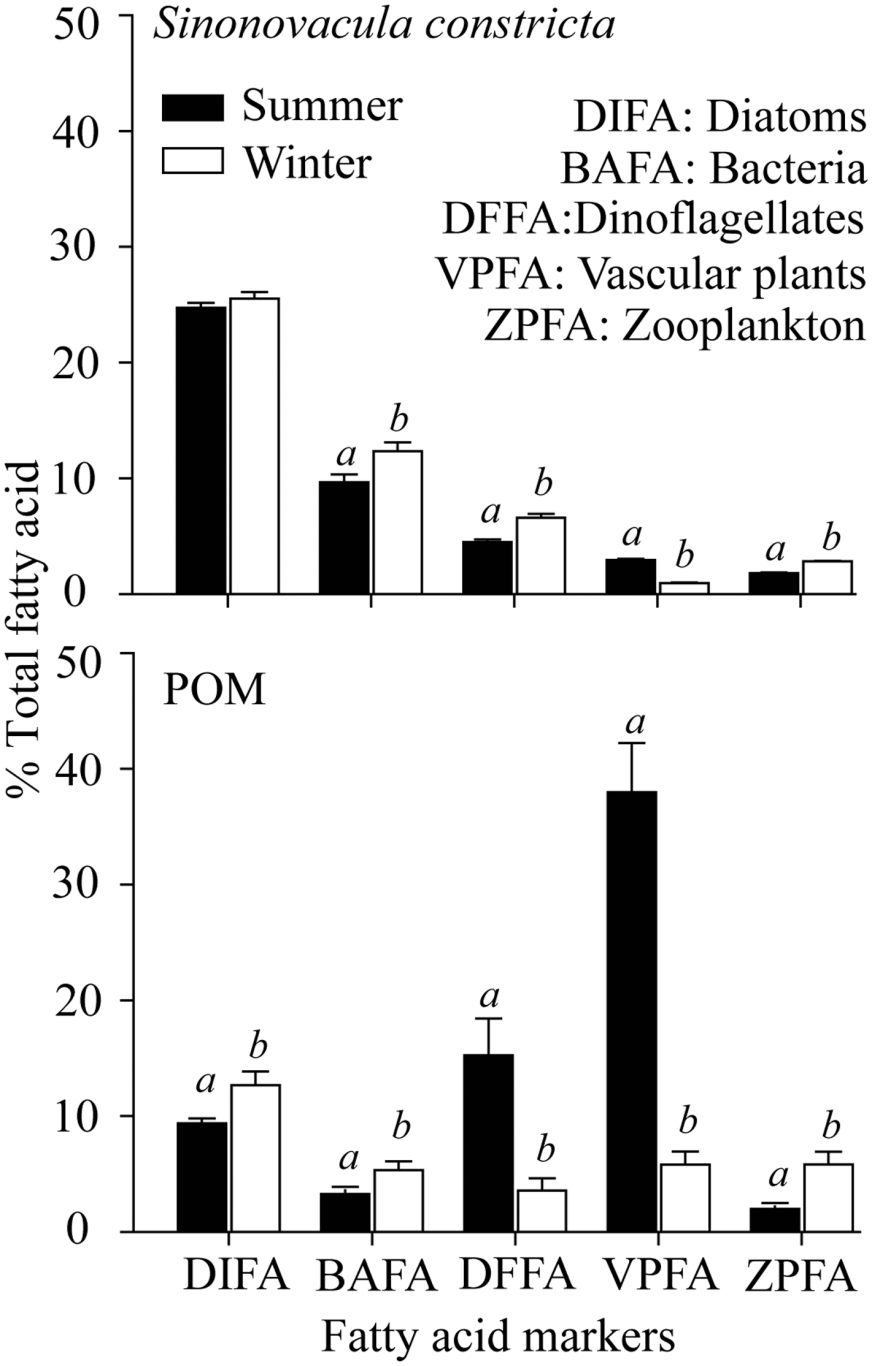
Relative percentages of fatty acid markers (diatoms, bacteria, dinoflagellates, vascular plants and zooplankton) from bivalve (*Sinonovacula constricta*) and POM in summer and winter. Different superscript letters (*a*, *b*) refer to significant differences between summer and winter.

Diatom and bacteria markers were enriched in the bivalve *S*. *constricta*, while dinoflagellate, vascular plant and zooplankton markers were relatively low (less than 10%) (Fig 6A). In this bivalve, the contribution of bacteria, dinoflagellate and zooplankton markers were significantly higher in winter compared with summer, however the relative contribution of plant markers was significantly higher during summer (Student’s *t*-test, *P* < 0.05).

Diatom, bacteria and zooplankton FA contributions to POM were significantly higher in winter compared with summer (Fig 6B). However the percentage of dinoflagellates and vascular plant markers were significantly higher in summer. The contribution of vascular plant FA markers to POM shows the largest change between summer and winter.

### Spatial differences in stable isotopes and fatty acids

#### Stable isotopes (SIs)

**Fig 7:**
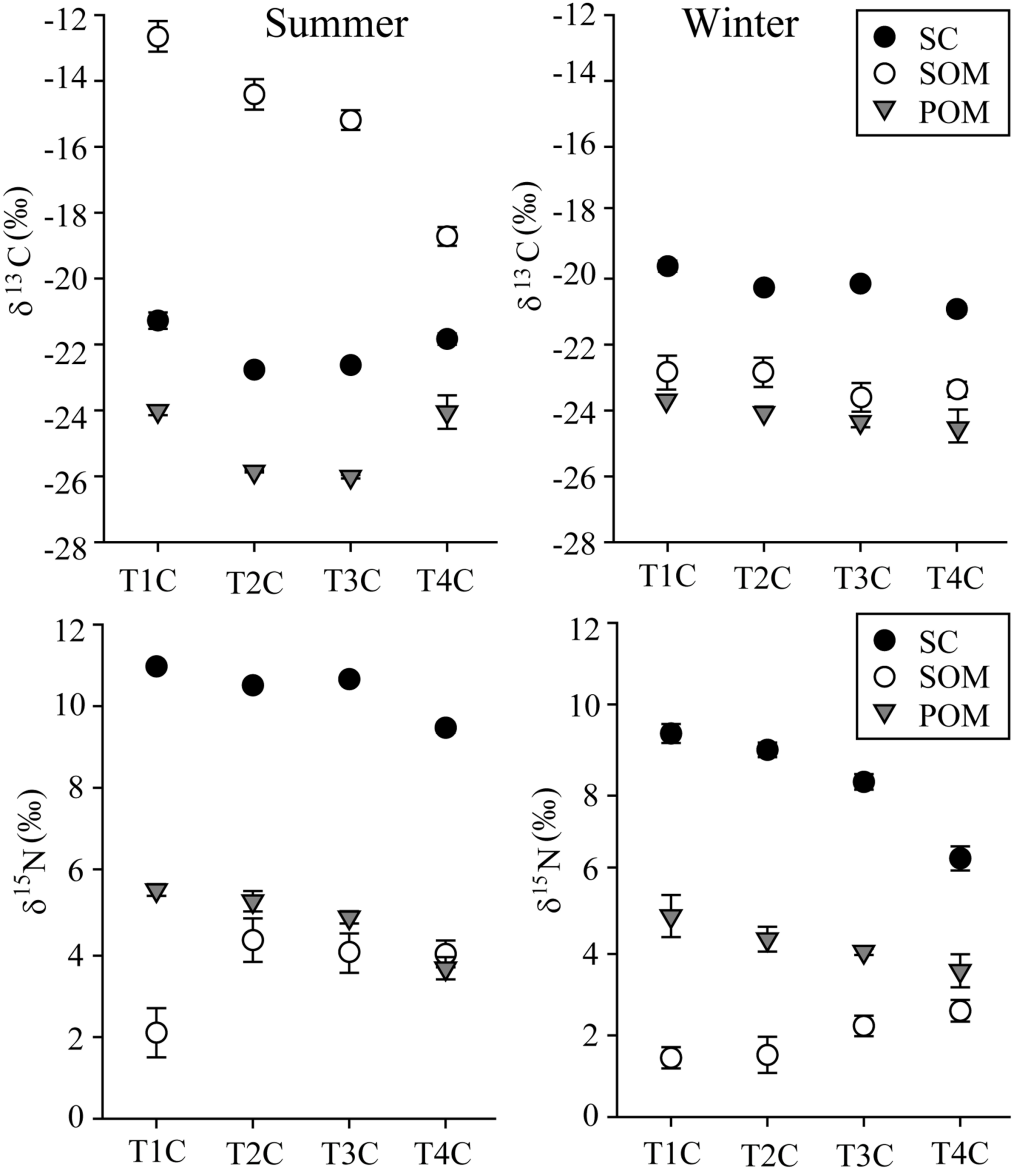
δ^13^C and δ^15^N of *Sinonovacula constricta* and SOM, POM from creek center sites of 4 transects in summer and winter.

In summer, the δ^13^C values for SOM decreased in transects with increasing distance from the main creek mouth (Fig 7). The δ^13^C values for POM and *S*. *constricta* in transects T2 and T3 were significantly lower compared with transects T1 and T4. In winter there was a general trend for decreasing δ^13^C values with distance from the creek mouth, but no significant differences were detected among the four transects.

The δ^15^N signatures for *S*. *constricta* decreased in transects with increasing distance from the creek mouth both in summer (from 9.57 to 10.98 ‰) and winter (from 6.28 to 9.42 ‰) (Fig 7). POM showed a similar pattern. The δ^15^N signatures for SOM were more enriched in transects T2 and T3 in summer, but there was a trend for increasing values with increasing distance from the creek mouth during winter.

#### Fatty acids (FAs)

**Fig 8:**
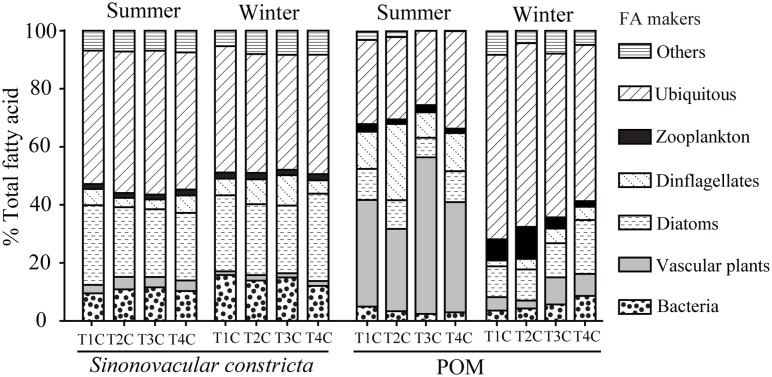
Fatty acid makers of bivalve *Sinonovacular constricta* and POM from creek center sites of 4 transects in summer and winter.

The relative contribution of each food group to FA markers composition in the bivalve, *S*. *constricta*, showed very little variation among the four transects (MANOVA, *F* = 1.34, *P* = 0.195) (Fig 8). There were no significant differences in the contribution of bacteria, vascular plants, diatoms, dinoflagellates and zooplankton markers to *S*. *constricta* among transects (Tukey HSD test, *P* > 0.05).

There were significant differences in the relative contributions of food groups to FA composition in POM among transects (MANOVA, F = 4.27, *P* < 0.0001) (Fig 8). In summer, the relative contributions of bacteria, diatoms, vascular plants and dinoflagellates were significantly different among transects (Tukey HSD test, *P* < 0.05). In winter, the relative contributions of vascular plants, diatoms and zooplankton markers varied significantly among transects (Tukey HSD test, *P* < 0.05).

## Discussion

### Ecological niche separation of different bivalve species

Two bivalve species identified in this study showed clear niche separation in spatial distribution at the Yangtze estuarine marsh. *Glauconome chinensis* dominated *Scirpus mariqueter* marshes in transects closest to the creek mouth. This is consistent with our previous findings that *G*. *chinensis* has a preference for *S*. *mariqueter* communities [42]. The bivalve species *Sinonovacula constricta* mainly occurred in the creek (Fig 2). The burrowing ability of different bivalve species into sediments of differing grain sizes, can affect their distribution [43]. *Sinonovacula constricta* has been found to inhabit deeper sediment layer than *G*. *chinensis* [44, 45]. Our field observation confirmed it and found that *S*. *constricta* mainly occur in creeks possess thicker soft top sediment. Previous studies also indicated that the distribution of benthic species was associated with tidal exposure time and sediment size, both of which might affect food availability to the benthos [46–49]. For instance, Sousa et al. (2008) indicated that the bivalve *Pisidium amnicum* distribution was related with different sediment characteristics and tidal influence [50]. In our study, *S*. *constricta* showed highest abundance in the creek at transect T2, probably due to the suitable currents and substrates at this site. For *G*. *chinensis*, its density showed no obvious variations among 5 marsh sites at T1, which suggests the distribution of this species was more closely related to elevation and plant types than to the distance to creek edge. Thus, many factors including the grain size of sediments, localized disturbances, hydrodynamics and plant type may account for the spatial segregation of bivalve species [51–53].

Distinct SI signatures suggest different carbon origins and trophic levels for the bivalves *S*. *constricta* and *G*. *chinensis* (Fig 3). The FA markers for diatoms, bacteria and vascular plants for the two bivalve species were also significantly different (Fig 4). This suggests that *S*. *constricta* and *G*. *chinensis* have separate dietary niches, though both are filter feeders. Variability in the resources available in different habitats may partly explain this difference. For instance, Kanaya et al. (2005) found the carbon source of *Ruditapes philippinarum* was mainly from suspended solids in its natural habitat, whereas when held in enclosures, the main carbon source was from benthic diatoms [54]. Morphological differences among bivalve species may also account for differences in their diet. For instance, *R*. *philippinarum* is a typical infaunal suspension feeder since it has a short inhalant siphon located at or below the sediment surface, while *Macoma contabulata*, a typical surface deposit feeder, mainly assimilates microphytobenthos as it extends a long inhalant siphon and is able to collect food items from the vicinity of the borrow during low tide [54]. For the species in our study area, *G*. *chinensis* lives in marshes and circulates water through its inhalant and exhalant siphons which are fused and only terminals separated [44, 55]. *Sinonovacula constricta* is mainly buried deeply in soft sediments of the creek. It inhales and exhales water via long and largely separated siphons [44, 56]. Therefore, the fact that *S*. *constricta* consume more diatom and bacteria and less plant materials than *G*. *chinensis* may be due to the differences of their microhabitat and morphology. These results show that site- and species-specific feeding habits should be considered when evaluating the roles of bivalves in transmitting materials in estuarine food webs.

### Food utilization and selection by bivalves

The δ^13^C values for sediment organic matter (SOM) were very close to the values for the plant *Spartina alterniflora* in summer (Fig 3), indicating that *S*. *alterniflora* could constitute a major source of carbon for SOM. This is consistent with the findings from a previous study in the same area which showed that SOM contained *S*. *alterniflora* derived C [57]. The increase in δ^13^C values for SOM at decreasing distances from the creek mouth during summer (Fig 7), could be attributed to the accumulation of *S*. *alterniflora* detritus during the summer flooding period. However, bivalves and SOM had significantly different δ^13^C signatures in this study, which implies that the *S*. *alterniflora* carbon source could not be highly assimilated by bivalves through SOM.

The higher δ^15^N values of *S*. *constricta* and *G*. *chinensis* than those of POM, probably reflecting trophic enrichment. Generally, the δ^15^N value is enriched by 3‒4 ‰ relative to its diet [26, 58]. This study showed 3.27‒5.79 ‰ δ^15^N enrichment between bivalves and POM, which indicates 1‒1.5 trophic level enrichment for bivalves compared to POM. Therefore, the bivalve may use POM as the main food item, and also could forage some zooplankton which consumed POM. In addition, bivalve and POM showed similar δ^13^C variation tendencies between different sites (Fig 7), indicating strong trophic links between bivalve and POM. This is consistent with the traditional view that POM is the principal food source for filter feeding infaunal bivalves [59–61]. For example, in the coastline of Southern Africa, the δ^13^C and δ^15^N signatures of the bivalve *Perna perna* changed with the POM [23]. In mangrove coral associated ecosystems in Fiji, the bivalve *Anadara* spp. mainly assimilate organic matter via POM [12]. In the Western Atlantic coast of Spain, Page et al. (2003) also found that bivalves (*Cerastoderma edule*, *Tapes decussatus*, *Mytilus galloprovincialis*) selectively ingest isotopically enriched components from the POM pool [9].

In estuarine salt marsh, POM may be derived from various sources including plant detritus, zooplankton, phytoplankton, macro/micro-algae and bacteria [62–64]. Our FA marker results indicated that plants, dinoflagellates, diatoms, bacteria and zooplankton were important components both of POM and in the diet of bivalves *S*. *constricta* and *G*. *chinensis* (Figs 4 and 6). The extent to which bivalves can select particles of higher nutritional value from POM has long been debated [65, 66], but it is generally accepted that many bivalves have the ability to assimilate particles selectively [60, 67–69]. For instance, Safi et al. (2007) found that *Atrina zelandica* preferentially selected algal species within the 2–20 μm size fraction, and this selection may be associated with particle morphotype, carbon content and potential toxicity [70]. In this study, we found that plants showed the greatest contribution (38.86 ± 4.25%) to POM in summer and that diatoms contributed the most (12.68 ± 1.17%) during winter (Fig 6). However, diatoms made the highest contribution to *S*. *constricta* both in summer (24.73 ± 0.44%) and winter (25.51± 0.59%), whereas plants contributed no more than 4%. In addition, dinoflagellates showed a higher contribution to POM in summer, and a higher contribution to *S*. *constricta* during winter. These seasonal differences further suggest that the bivalve *S*. *constricta* has an assimilation selectivity when they ingest organic matter from POM.

Feeding selectivity has a profound impact at both the individual and ecosystem levels and could serve as an important mechanism limiting resource competition between species through trophic plasticity. Filter feeding of bivalves can have a large impact on both the benthic and pelagic compartments of the ecosystem. Many ecologists have shown that different bivalve species modify sediment properties, phytoplankton, bacterial communities and microphytobenthic biomass differently which were associated with their feeding ecologies [7, 29, 71–73]. In addition, selective feeding can also change nutrient cycling at the ecosystem scale [7]. It has been reported that suspension-feeding bivalves are able to sort captured particles, ingesting particles of high nutritional value and rejecting those of low nutritional value (e.g., particulate inorganic matter and refractory detritus), and thus to change the nutrient pool [7, 74]. Therefore, the study of interactions between organisms and the environment requires a certain level of detail concerning the feeding process, not only from the organism point of view (which material can they actually use as food) but also from the ecosystem point of view (to what extent are organisms able to change ecosystem dynamics) [11].

### Spatial and temporal changes in food resources of the bivalve *Sinonovacula constricta*

Previous studies have demonstrated the primary importance of phytoplankton (notably diatoms and seasonally abundant dinoflagellates), zooplankton and microphytobenthic material in the diets of suspension feeding bivalves [8, 9, 34, 59]. Spatial and temporal fluctuations in the relative contributions of various sources of organic matter to bivalves, have also been observed [6, 16, 23].

Changing isotope values of consumers are generally associated with changes in the SI values of food sources [75]. The process of microbial degradation of dead plants should result in an overall decrease in δ^15^N values in winter [76]. In this study, δ^15^N of all food sources were more depleted in winter than during summer, leading to a similar change in the δ^15^N signature of the bivalve *S*. *constricta*. Phanerogam degradation can lead to a ^13^C-depletion possibly due to higher levels of lignin which is ^13^C-depleted in winter [36, 77]. This probably explains why δ^13^C values for plants and SOM were lower in winter than during summer in our study. However, δ^13^C values for POM were higher during winter than in summer (Fig 3). Carbon enrichment of POM in winter could indicate temporal changes in the near shore detrital pool, presumably reflecting changes in algal detritus and links to hydrography and algal seasonality [78]. FAs in our study confirmed it that POM contains a proportional greater amount of plant materials in summer and more diatoms in winter (Fig 6). The synchronous seasonal variations for *S*. *constricta* and POM implied that the changes in δ^13^C values for *S*. *constricta* were mainly due to the changes in POM values. Previous studies have also recorded ^13^C depletion of the bivalve (*Gryphaea* (*Bilobissa*) *dilobotes*) occurring during warmer periods, possibly related to an interaction between plankton blooms and intra-annual variations in mixing across a thermocline [79]. However Lebreton et al. (2011) found that *Cerastoderma edule* and *Tapes phillipinarum* exhibited higher δ^13^C values in summer than in winter [36]. Thus seasonal alternations in the carbon source composition of bivalves may be affected by different factors and probably rely on changes of carbon composition in POM.

The FA composition of *S*. *constricta* provided insight into temporal variation in its diet. Diatoms were the most important food source for *S*. *constricta* during both summer and winter (Fig 6). The contribution of bacteria, dinoflagellates and zooplankton to the diet of *S*. *constricta* were greater in winter than in summer, but plant detritus made a higher contribution during summer. Consistent with the SI results, FA composition also suggested that there are seasonal changes in food availability and nutrient requirement for *S*. *constricta* in the Yangtze River estuary. Similar seasonal changes in food composition for bivalves have been reported. At Tomigusuku intertidal flat of Okinawa Island (Japan), the primary food components (vascular plants, bacteria, macroalgae and phytoplankton) of the bivalve *Quidnipagus palatum* vary seasonally [17].

Consumers in all regions relied most heavily on locally produced organic matter. For example, Deegan et al. (1997) evaluated organic matter to the estuarine food web of Plum Island Sound, Massachusetts, USA [3]. They found most consumers in the upper estuary had δ^13^C values of ‒29 to ‒21‰, which indicated dependence on a mixture of fresh marsh emergent vegetation and phytoplankton, while in the middle and lower estuary, consumers had δ^13^C values resembling a mixture of *Spartina* spp., benthic microalgae and marine phytoplankton [3]. In the Yangtze River estuary of this study, spatial variation in the FA profiles and SI signatures for POM indicated that the availability of food sources differed greatly among transects at different elevations along the tidal creek (Fig 8). Comparing to POM, however, the spatial variations in FA profiles for *S*. *constricta* were much smaller. This difference in FA profiles for POM and *S*. *constricta* suggests the selectively feeding of the bivalve. Nevertheless, the SI value variations for *S*. *constricta* changed similarly with POM among transects (Fig 7). The bivalve *S*. *constricta* inhabiting the creek mouth had relatively higher δ^13^C values than in the upper creek, which indicates that it may absorb more carbon source from the exotic C_4_ plant *S*. *alterniflora*. While in the upper creek area, carbon source of *S*. *constricta* may derive mainly from native C_3_ plants *P*. *australis* and *S*. *mariqueter*. Previous study also showed spatial shifts in carbon sources for bivalves. Doi et al. (2005) found δ^13^C value of the bivalve *Corbicula japonica* significantly decreased from down-stream to up-stream in the Kitakami River, northeastern Japan, which correlates with the changing contributions of marine and terrestrial organic material to the POM [16]. The oyster *Crassostrea gigas* exhibited significantly different δ^13^C variations between sites along an estuarine gradient, probably because it absorbed more terrestrial organic matter in the upper estuary [80]. This may indicate that although the bivalve had a similar diet composition at each of the different sites, these diets were derived from different carbon sources. Therefore the changes in the composition of organic matter with distance from the mouth of the marsh creek, and the selective feeding habits of bivalves, will in turn lead to spatially heterogeneous food webs in estuarine marshes.

In this study, SI and FA techniques were integrated to reveal trophic dynamic information of suspending feeding bivalves, which are difficult to study with classical dietary analysis methods. SI and FA methods are highly informative in tracing the fate of primary production through detrital and other pathways in such complex estuarial salt marsh environments. However, the results still should be interpreted with caution, because SI values and FA markers can vary greatly with habitats and/or organisms. For instance, long chain FAs can serve as indicators of vascular plants in SOM and POM, but their usage as tracers in animal food webs is limited because they are not assimilated by some consumers [81]. In terms of SI, the δ^15^N enrichment between consumer and its diet can vary with different habitats. Organisms inhabiting marine environments yielded significantly lower δ^15^N enrichment than those from terrestrial or freshwater systems [82]. In addition, benthic communities in estuaries may contain more potential food sources than we assume and can detect with FAs [81]. Therefore, more analyses by using multi-approaches including field sampling, controlled feeding experiments, multivariate and univariate analyses will be helpful to yield more concrete results.

## Conclusion

Based on distribution patterns and food sources, our study indicates that the different bivalve species examined in this study, had separate ecological niches in the Yangtze estuarine marshes. Both bivalve, *S*. *constricta* and *G*. *chinensis* mainly ingested organic matter from POM. Diatoms, bacteria, dinoflagellates, vascular plants and zooplankton formed major components of the diet of bivalves. Although the contribution of diatoms to the diet of *S*. *constricta* was consistent, the relative contribution of other food items varied between summer and winter, indicating seasonal changes in food availability. The seasonal variation patterns in item composition between *S*. *constricta* and POM suggest that the bivalve has an assimilation selectivity when they ingest organic matter from POM. Constant FA profiles and varied SI values indicated that *S*. *constricta* had a similar diet at different sites, although the material was derived from different carbon origins. It is suggested that species-specific and temporal and/or spatial variability in the feeding of bivalves can affect their ecological functions in intertidal marshes. This should be considered in the study of food web dynamics and the flow of material in estuarine ecosystems.

## Supporting Information

S1 Table Elevation (cm) and plant communities at different sites of 4 transects. (DOCX)

S2 Table Fatty acid composition (% of total fatty acids) of the bivalve (*Sinonovacula constricta*, *Glauconome chinensis*) and food sources (SOM, POM, *Phragmites australis*, *Spartina alterniflora*, *Scirpus mariqueter*) from the Yangtze River estuarine Jiuduansha salt marsh in summer and winter. (DOCX)
