# Supplementary material for: Trophic Dynamics of Filter Feeding Bivalves in the Yangtze Estuarine Intertidal Marsh: Stable Isotope and Fatty Acid Analyses
Source: PLoS One. 2015 Aug 11;10(8):e0135604. doi: 10.1371/journal.pone.0135604 (PMC4532420; doi:10.1371/journal.pone.0135604)
Supplement: S1 Table — (DOCX) [file pone.0135604.s001.docx]

**S1 Table. Elevation (cm) and plant communities at different sites of 4 transects.**

| Sites | Transect 1 | | Transect 2 | | Transect 3 | | Transect 4 | |
| --- | --- | --- | --- | --- | --- | --- | --- | --- |
|  | Elevation | Plant | Elevation | Plant | Elevation | Plant | Elevation | Plant |
| Creek center | 138 | — | 159 | — | 197 | — | 257 | — |
| Creek edge | 391 | — | 452 | — | 461 | — | 470 | — |
| 1m from creek bank | 436 | *SM* | 499 | *SA* | 494 | *SM* | 482 | *PA* |
| 5m from creek bank | 431 | *SM* | 574 | *SA* | 540 | *SM* | 496 | *PA* |
| 10m from creek bank | 431 | *SM* | 575 | *SA* | 562 | *SA* | 495 | *PA* |
| 25m from creek bank | 435 | *SM* | 572 | *SA* | 547 | *SA* | 489 | *PA* |
| 100m from creek bank | 441 | *SM* | 574 | *SA* | 524 | *PA* | 484 | *PA* |

*SM*, *SA*, *PA* represent the plant type of *Scirpus mariqueter*, *Spartina alterniflora* and *Phragmites australis* respectively.
