# Supplementary material for: Trophic Dynamics of Filter Feeding Bivalves in the Yangtze Estuarine Intertidal Marsh: Stable Isotope and Fatty Acid Analyses
Source: PLoS One. 2015 Aug 11;10(8):e0135604. doi: 10.1371/journal.pone.0135604 (PMC4532420; doi:10.1371/journal.pone.0135604)
Supplement: S2 Table — (DOCX) [file pone.0135604.s002.docx]

**S2 Table. Fatty acid composition (% of total fatty acids) of the bivalve (*Sinonovacula constricta*, *Glauconome chinensis*) and food sources (SOM, POM, *Phragmites australis*, *Spartina alterniﬂora*, *Scirpus mariqueter*) from the Yangtze River estuarine Jiuduansha salt marsh in summer and winter.**

|  | *S. constricta* | | *G. chinensis* | SOM | | POM | | *P. australis* | *S. alterniflora* | *S. mariqueter* |
| --- | --- | --- | --- | --- | --- | --- | --- | --- | --- | --- |
| Fatty acids | Summer | Winter | Summer | Summer | Winter | Summer | Winter | Summer | Summer | Summer |
| **C14:0** | 7.01±0.14 | 9.89±0.28 | 5.63±1.7 | 3.69±0.56 | **11.31±0.64** | 5.48±0.21 | 9.37±0.63 | 1.37±0.21 | 1.41±0.23 | 0.84±0.09 |
| **C14:1** | 0.44±0.05 | 1.09±0.29 | 0.63±0.15 | 0±0 | 1.08±0.11 | 0±0 | 2.31±0.4 | 0±0 | 0.28±0.12 | 0.26±0.06 |
| **C15:0** | 3.68±0.08 | 5.44±0.2 | 2.51±0.36 | 1.16±0.1 | 2.91±0.15 | 2.08±0.3 | 2.27±0.29 | 0.63±0.14 | 0.38±0.05 | 0.89±0.09 |
| **C16:0** | **35.63±0.26** | **26.35±0.36** | **37.61±0.24** | **17.55±1.52** | **30.81±1.04** | **20.97±0.63** | **35.93±1.56** | **33.53±2.68** | **28.68±1.25** | **34.27±2.32** |
| **C16:1** | **13.05±0.34** | **10.61±0.54** | 8.29±4.23 | 3.37±0.81 | 8.87±2.14 | 3.5±0.25 | 2.71±0.54 | 2.8±0.31 | 3.93±0.56 | 2.98±0.21 |
| **C17:0** | 2.19±0.02 | 2.68±0.06 | 2.82±0.51 | 0.52±0.1 | 1.01±0.14 | 0.63±0.06 | 1.12±0.18 | 0.46±0.04 | 0.21±0.09 | 0.45±0.06 |
| **C17:1** | 0.54±0.04 | 0.98±0.07 | 0.55±0.1 | 0±0 | 5.46±1.14 | 0±0 | 1.26±0.33 | 0±0 | 0±0 | 0±0 |
| **C16:4n3** | 2.18±0.16 | 2.33±0.17 | 1.84±0.75 | **19.35±2.98** | 1.97±0.44 | **15.24±3.18** | 3.24±0.96 | 0±0 | 0±0 | 0±0 |
| **C18:0** | 6.76±0.14 | 9.95±0.21 | 8.73±1.85 | 2.65±0.21 | 7.26±0.85 | 4.08±0.24 | 8.88±0.78 | 1.85±0.19 | 1.55±0.07 | 1.47±0.15 |
| **C18:1n9** | 5.6±0.17 | 5.84±0.14 | 3.55±0.17 | 2.36±0.32 | 6.62±0.97 | 5.68±0.77 | **15.75±0.99** | 1.5±0.51 | 2.73±0.5 | 3.55±0.15 |
| **C18:1n7** | 4.54±0.07 | 4.07±0.13 | 4.05±0.72 | 0.4±0.05 | 1.4±0.25 | 0.54±0.06 | 0.47±0.12 | 0.46±0.46 | 0.31±0.18 | 0±0 |
| **C18:2n6** | 1.96±0.08 | 0.67±0.02 | 1.87±0.02 | 1.38±0.11 | 1.52±0.16 | 1.71±0.11 | 2.4±0.43 | **12.53±0.91** | **16.41±1.37** | **13.24±0.9** |
| **C18:3n6** | 0.47±0.01 | 0.32±0.03 | 0.66±0 | 0±0 | 0±0 | 0±0 | 0±0 | 0±0 | 0.19±0.08 | 0±0 |
| **C18:3n3** | 1.27±0.03 | 0.51±0.04 | 3.01±0.41 | **45.41±3.86** | 4.48±0.61 | **36.81±4.29** | 3.42±0.6 | **40.31±3.52** | **39.74±2** | **37.74±2.13** |
| **C18:4n3** | 0.67±0.02 | 0.75±0.02 | 0.83±0 | 0±0 | 0.44±0.08 | 0±0 | 0.2±0.12 | 0±0 | 0±0 | 0±0 |
| **C20:0** | 0.49±0.03 | 0.8±0.05 | 0.32±0 | 0.21±0.04 | 1.31±0.23 | 0.17±0.07 | 0.61±0.21 | 0.33±0.07 | 0.28±0.09 | 0.22±0.05 |
| **C20:1** | 1.36±0.04 | 1.25±0.03 | 1.66±0.06 | 0.09±0.04 | 0.38±0.1 | 0.22±0.11 | 3.3±1.31 | 0±0 | 0±0 | 0±0 |
| **C20:2** | 1.08±0.02 | 0.7±0.02 | 0.96±0.35 | 0±0 | 0.06±0.06 | 0±0 | 0.29±0.29 | 0±0 | 0.07±0.02 | 0±0 |
| **C20:3n6** | 0.11±0.02 | 0±0 | 0.17±0 | 0±0 | 0±0 | 0±0 | 0±0 | 0±0 | 0.07±0.03 | 0±0 |
| **C20:4n6** | 2.04±0.1 | 3.37±0.17 | 4.79±2.48 | 0.18±0.03 | 0±0 | 0.17±0.06 | 0±0 | 1.08±1.02 | 0.28±0.13 | 0±0 |
| **C20:3n3** | 0±0 | 0±0 | 0.29±0 | 0.16±0.03 | 0.79±0.19 | 0.11±0.06 | 1.24±0.25 | 0.07±0.05 | 0.17±0.13 | 0.08±0.02 |
| **C21:0** | 0.15±0 | 0±0 | 0.17±0 | 0±0 | 0±0 | 0±0 | 0±0 | 0±0 | 0±0 | 0±0 |
| **C20:5n3** | 4.67±0.16 | 5.01±0.21 | 5.59±1.18 | 0.44±0.12 | 1.32±0.4 | 0.37±0.09 | 0.6±0.32 | 0.16±0.06 | 0.44±0.21 | 0.11±0.04 |
| **C22:0** | 0.15±0.01 | 0.26±0.03 | 0.17±0 | 0.29±0.04 | 1.41±0.19 | 0.23±0.06 | 0.96±0.26 | 0.2±0.07 | 0.18±0.08 | 0.15±0.04 |
| **C22:1n9** | 0.44±0.02 | 0.98±0.03 | 0.2±0 | 0.43±0.06 | 7.39±1.17 | 1.76±0.18 | 2.53±0.75 | 0±0 | 0±0 | 0±0 |
| **C22:2** | 0.11±0.02 | 0.43±0.03 | 0±0 | 0±0 | 0±0 | 0±0 | 0±0 | 0±0 | 0±0 | 0±0 |
| **C23:0** | 0.76±0.05 | 1.23±0.06 | 1.03±0.23 | 0.06±0.02 | 0.47±0.12 | 0±0 | 0.2±0.11 | 0.08±0.02 | 0±0 | 0.13±0.05 |
| **C22:6n3** | 2.51±0.13 | 4.39±0.23 | 3.47±0.66 | 0.05±0.02 | 0.13±0.06 | 0±0 | 0.13±0.07 | 0±0 | 0±0 | 0±0 |
| **C24:0** | 0.15±0.04 | 0.08±0.03 | 0±0 | 0.14±0.04 | 1.28±0.17 | 0.12±0.04 | 0.2±0.09 | 0.29±0.09 | 0.11±0.07 | 0.4±0.14 |
| **C24:1** | 0±0 | 0±0 | 0±0 | 0.11±0.04 | 0.23±0.2 | 0.11±0.05 | 0.56±0.37 | 0±0 | 0±0 | 0±0 |
| ∑SFA | 56.98±0.3 | 56.69±0.45 | 59±0.45 | 26.27±2.3 | 57.76±1.61 | 33.77±1.1 | 59.55±1.59 | 38.74±3.16 | 32.79±1.53 | 38.82±2.85 |
| ∑MUFA | 25.96±0.26 | 24.82±0.82 | 18.93±5.32 | 6.76±0.92 | 31.44±1.89 | 11.82±0.95 | 28.9±1.12 | 4.8±0.37 | 7.33±1.18 | 6.81±0.37 |
| ∑PUFA | 17.09±0.45 | 18.48±0.72 | 23.48±4.87 | 66.97±3.18 | 10.76±0.83 | 54.41±1.85 | 11.51±1.55 | 54.21±3.16 | 57.38±1.69 | 51.2±2.73 |

Data are shown as mean ± SE. Only fatty acids >0.05% are shown, with the numbers in bold indicating >10% contribution.

SFA, saturated fatty acid; MUFA, monounsaturated fatty acid; PUFA, polyunsaturated fatty acid. SA and PA represent samples from *S. alterniﬂora* and *P. australis* habitat, respectively. SOM: sediment organic matter; POM: particular organic matter.

No *G. chinensis* fatty acid data in winter, because there are too less sample quantity of *G. chinensis* to fatty acid analysis.
